# Prognosis Factors in Chronic Lymphocytic Leukemia

**Published:** 2012

**Authors:** AM Ivanescu, M Oprea, R Momanu, E Coles, A Colita, AR Lupu

**Affiliations:** * Hematology Department, Coltea Clinical Hospital, Bucharest; ** Roche Romania, Bucharest; *** “Carol Davila” University of Medicine and Pharmacy, Bucharest

**Keywords:** Chronic lymphocytic leukemia, prognosis factors, CD38, ZAP70, IgHV, CLL - Chronic lymphocytic leukemia, LDT - lymphocyte doubling time, CD38 - cluster of differentiation 38, ZAP-70 the protein associated to zeta chain with the molecular weight of 70Kda, NK - natural killer cells, IGHV - immunoglobulin heavy chain variable region genes

## Abstract

Chronic lymphocytic leukemia is still one of the most common hematologic malignancies. Finding a curative solution is the objective of numerous followed cases and clinical trials. Diagnosis is based on the interlocking of classic elements and newly identified prognostic factors but time to first treatment is still an open issue. CD38, ZAP 70, IgHV gene mutational status and cytogenetic changes are proven negatively influence the evolution of chronic lymphocytic leukemia. Whether through aggressive rapid evolution or by the difficulty of obtaining a complete remission or risk of early relapse, CLL is still important. Adapted to these prognostic factors, combined therapeutic regimens have proved to be effective in achieving a durable complete remission, new agents, with encouraging partial results, being studied. Requiring initial screening, for comparative purposes, a current and growing importance has minimal residual disease; its absence at the end of treatment represents a strong positive prognostic factor.

## Introduction

Chronic lymphocytic leukemia (CLL) is a lymphoproliferative disease characterized by the accumulation of small lymphocytes apparently morphologically mature, but biologically immature, in bone marrow, lymph nodes, blood, spleen, liver and sometimes in the other organs. In most cases, it is about the clonal expansion of B line, less common being the cases in which tumor cells have T phenotype. As the most common form of leukemia in about 30% of cases [**1**], and diagnosed at a medium age between 65-70 years [**2**], CLL is seen in a proportion of 20-30% of cases in people less than 55 years old [**3**]. CLL is considered a hematologic malignancy with a highly heterogeneous trend and there are cases of aggressive and rapidly evolving situations, but also cases with decades of survival [**4**], the average survival ranging between 5 and 10 years [**5**]. Young patient survival period is still frequently limited by complications related to the disease or within the treatment. It is also known that not all patients require chemotherapy from the diagnosis. The identification of the prognosis elements which are imposed the initiation of treatment since the diagnosis, represents the key of success in achieving a long-term remission.

## Factors of prognosis

Following to the tests and studies that included patients diagnosed with CLL, it has been attempted the correlation of clinical and paraclinical elements with unfavorable evolution of the cases. Thus have been found a series of unfavorable prognosis factors whose evaluation may be initiated at diagnosis moment of the patient, but may be made throughout evolution, especially in cases where the disease is refractory or progressive or in the situation of disease relapse. Classic prognosis factors, traditionally, are known, and with the emerge and improvement of modern investigative methods have thus created new guidelines for diagnosis, were added recent risk factors with importance in the evolution, prognosis and optimal treatment choice for patients diagnosed with CLL [**6**] (**Table 1**).

**Table 1.** Classical and biological prognostic markers of CLL. Modified after: Furman RR (2010) Prognostic markers and stratification of chronic lymphocytic leukemia. Hematology Am Soc Hematol Educ Program 2010:77–81[**7**]

- Classical prognostic markers
Clinical stages
• Lymphocytes morphology in peripheral blood
• Blood lymphocyte count
• Blood lymphocyte doubling time
• Bone marrow infiltration degree (biopsy/aspirate)
• Biological prognostic markers
- Serum markers
• tymidine-kinase
• bete-2 microglobulin
• soluble CD23
V3-21 gene usage
IgHV mutational status
FISH cytogenetics
High risk: +12,11q-, 17p-, complex karyotype
Low risk: normal cariotipe, 13q-
CD38 expression
ZAP 70 expression
Further studies required
miR signature, CLLU1 expression
chromosomal translocations
TCL-1 gese, MCL-1 expression, Bcl-2/Bax ratio
MDR1/MDR3 genes
Lipoprotein lipase A expression
Activation indeced cytidine deaminase mRNA
VEGF, ADAM 29 expression
Thrombopoietin
Telomere length and telomerase activity
CD49d, CD69, FCRL
- Treatment related markers
Response to therapy (minimal residual desease)

**Stadialisation of the patients** with CLL is realized based on the 2 classifications widely recognized. Rai Classification [**8**] includes Stage 0: - peripheral lymphocytosis> 15.000/µl, medullary lymphocytosis > 40%, Stage I: - stage 0 + palpable adenopathies; Stage II - peripheral lymphocytosis> 15.000/µl, medullary lymphocytosis > 40% + hepatomegaly and / or splenomegaly ± adenopathies; Stage III - peripheral lymphocytosis> 15.000/µl, medullary lymphocytosis > 40% + anemia (Hb <11g/dL) ± adenopathies ± splenomegaly / hepatomegaly; Stage IV - peripheral lymphocytosis> 15,000 / ml, medullary lymphocytosis > 40% + thrombocytopenia (platelets no. < 100.000/µl) with or without remaining elements mentioned above.

Binet and collaborators [**9**] have developed a new classification according to the stadium. This takes into account that there are 5 lymphatic areas: cervical lymph nodes, inguinal lymph nodes, axillary lymph nodes, spleen and liver. Binet defines three stages: A Stage: - lymphocytosis + less than three interested lymph areas; B Stage: - lymphocytosis + more than three interested areas; C Stage: - lymphocytosis + anemia and / or thrombocytopenia.

Among the **classical prognosis factors**, lymphocyte-doubling time (LDT) is a useful method for measuring disease progression. LDT is defined as the number of months required for absolute lymphocyte count to double. Patients with a short LDT (≤ 12 months) have a survival, total and without treatment, significantly shorter than those with longer lymphocyte doubling time. Independent of disease stage/stadium, the average survival of patients with LDT shorter than 12 months, is significantly lower than those with LDT longer than 1 year.

However, the use of LDT requires repeated assessments of patients and is inherently retrospective. Also, the number of lymphocytes may be influenced by factors other than disease progression, requiring more than two or three longitudinal assessments. Additionally, the patients may have discordant progressive disease, compared to the rate of progression of lymphocytes. For example, some patients may have a relatively stable number of lymphocytes, but leukemic cells with a formation rate to 1% are detected from the total of the leukemic clone per day. LDT significance should take into account the clinical context of the patient altogether [**10**]. With regard to certain serum factors, in the conditions of a normal renal function, some serum proteins may occur in high concentrations in patients with aggressive disease. Moreover, the relative level of each of these proteins was correlated with the kinetics of tumor progression. There are important reserves in the use of these serum markers, because their levels can increase over time with increased tumor burden and may be altered in case of renal failure. Some treatments, diseases or renal dysfunction may affect the relative level of these factors, compromising their use as prognostic factors [**11,12**].

**CD38.** The patients with aggressive disease, often malignant lymphocytes expressing CD38, a transmembrane glycoprotein of 45 KDa, able to synthesize cyclic riboso-adenosine diphosphate (riboso ADP) from nicotinamide adenosine dinucleotide and to hydrolyze cyclic riboso-ADP to riboso-ADP. Several corroborated studies have concluded that CD38 is an indicator of unfavorable prognostic, independent of clinical stage. Increased expression of CD38 remains associated with a negative prognosis, although probably not have the same ability to predict the prognosis against IgHV genes expression without mutations, or other prognostic markers such as protein of 70Kda associated to zeta chain (ZAP-70). CD38 usage, as a prognostic indicator, may be influenced by technical differences used to differentiate "positive" cases versus "negative" ones of CD38. Such ZAP-70, the distribution of CD38 expression levels in different populations of patients, fails to clearly define 2 subgroups of patients. For this reason, it is necessary to define "positive" cases according to the assessment of leukemic cells as having fluorescence intensity > 30% above the threshold considered as positive [**13-18**].

### ZAP-70 (the protein associated to zeta chain with the molecular weight of 70KDa)

B-lymphocytes in CLL, that do not express mutations in IgHV genes, can be differentiated from those that express different mutations by expression of a relatively small subset of genes. One of these genes encodes ZAP-70; it is a cytoplasm tyrosine- kinase weighing 70KDa which normally is expressed only in the natural killer cells (NK) and T lymphocytes, where has the ability to associate with CD247, the CD3 zeta chain (CD3ζ-chain) from the level of the T cell receptor. By the contrast to leukemic B lymphocytes showing receptor gene mutations, in cells using IgHV genes without mutations, express RNA ZAP-70. Some studies have discovered that B cells from CLL without mutations, present generally, the levels of ZAP-70 protein comparable to those expressed by normal circulating T lymphocytes. On the other hand, leukemic cells having mutations in IgHV gene do not express detectable levels of ZAP-70 protein. ZAP-70 expression in CLL cells can be used as a marker for mutagenic status of immunoglobulins, this indicator can separate the patients who have significantly different trends of disease progression [**13,19,20**].

Using sensitive flow cytometry techniques, distribution of ZAP-70 protein expression levels in different leukemia patients cannot clearly define two subpopulations. Therefore, it is necessary to define “positive” cases based on leukemia cells with fluorescence intensity ≥ 20% above the threshold considered positive, the limit that has clinical significance [**15**].

Although malignant lymphocytes in CLL using IgHV genes without mutations express, also, ZAP-70, this is not a valid rule for all cases. In turn, there are cells that express ZAP-70, although they have mutations in IgHV genes. An extensive study realized in several institutions, observed that patients with positive ZAP-70 cells have an average duration, from diagnosis to initiation of therapy by 2.8 years, if IgHV cells express genes without mutations, and by 4.2 years, if genes have mutations. These two subpopulations of patients presented no significant differences. However, the average time from diagnosis to initiation of therapy in these two groups was significantly shorter than in patients with negative ZAP-70 cells, which presented or not, mutations in IgHV genes (P <0.001). The average duration from diagnosis to initial therapy in this group of patients was 11 years, for those with mutations in IgHV genes, and 7.1 years for those without the gene mutations (P <0.001). It can be concluded that the expression of ZAP-70 in leukemic cells in B-CLL is a strong prognostic factor for early treatment indication than mutagenic status of IgHV [**20**].

Additional studies have found that ZAP-70 has functional significance in chronic lymphocytic leukemia. It can intensify the signaling capacity of surface Ig, expressed in CLL; this function is apparently independent from its own kinase activity. This can allow to positive ZAP-70 cells to obtain more stimulation from self-or non-self-antigens, which interact with selected immunoglobulins used in CLL. At a repeated exposure to such antigens, the leukemia B-lymphocytes can get a high stimulation, leading to increased proliferation and / or resistance to apoptosis. Thus, ZAP-70 expression may be more closely correlated with the tendency of early disease progression than mutagenic status of IgHV gene. According to this notion, there are clinical investigations, which have concluded that ZAP-70 expression in CLL cells is a stronger predictor of aggressive disease than CD38+ cells existence or IgHV genes without mutations [**20,21**].

**Genetic mutations in the immunoglobulins.** Mutation status of Ig genes expressed by CLL cells can be used to separate patients into two subsets, which have significantly different trends in the disease. Cells that do not express mutations in IgHV genes, may have trisomy 12 and atypical morphology more frequently than those expressing mutations but, instead, tend to have more frequent abnormalities involving chromosome 13 (13q14). Furthermore, patients with leukemia cells without IgHV genes mutations have a greater tendency to disease progression than those expressing IgHV genes with the nucleic acid sequence homology with germline, less than 98%, the observation confirmed by sequential studies. Although the mutagenic status of immunoglobulins does not seem to influence the relative response to treatment, patients with malignant cells without expressed IgHV genes mutations, appear to have a significantly shorter duration of remission than those with mutations in these genes. An exception to this fact is represented by patients whose leukemia cells use a particular gene of immunoglobulins, for example IGHV3-21. This gene can present somatic mutations when it is expressed by B-lymphocytes in chronic lymphocytic leukemia. However, the patients with leukemia cells that use a IGHV3-21 genes with mutations, together with immunoglobulins with λ light chain encoded by IGHV3-21 genes, present a risk of aggressive disease, similarly to the patients with cells expressing IgHV genes with no mutations [**2,13,19,22**].

**Elements of cytogenetics**. It has been demonstrated that certain specific chromosomal abnormalities have clinical significance, in prognosis and pathogenesis of several hematologic malignant diseases. The focus was mainly on acute leukemia and chronic myeloid leukemia. In case of CLL, the most common chromosomal abnormality is 13q deletion (del 13q) [**19,23-25**]. Using a battery of 6 microsatellite markers from 13q12.3 to 14.3 between BRACA2 gene and Rb gene, 13q14 deletion was found in leukemia cells of 29 patients from 78 patients with CLL [**26**]. However, the classical cytogenetics seems to have a lower sensitivity because such method to detect 13q14 deletion in leukemic cells from 1% of cases. The patients with CLL who have 13q14 deletion have a poor prognosis compared to those without this abnormality. At the same time, patients with del 13q14 seem to have a better prognosis than those with trisomy 12.

Trisomy 12 is the following chromosomal abnormality found with a high frequency. Approximately one per third of patients have chromosome 12 as the only abnormality. Some studies [**27**] have identified 12 trisomy in progenitor CD34 cells + from bone marrow or blood, in a subset of patients with CLL. Nevertheless, subsequent researches indicated that the subset of patients with CLL, with aberrant CD34 + cells from genetically point of view, is extremely rare [**28**]. Therefore, chronic lymphocytic leukemia is, generally, not associated with genetic defects in pluripotent stem cells. Instead, trisomy 12 seems to be a genetic abnormality acquired during the progress of the disease [**29**]. Nevertheless, a longitudinal study conducted on 41 patients, found no significant changes in the relative proportion of cells with trisomy 12 within 4 years, even in patients with progressive disease [**30**].

Analyzing simultaneously, cell morphology, immunophenotyping and karyotyping of the same cell, thus it has been demonstrated that Trisomy 12 occurs in neoplasic B cells, but not in those of normal T type [**31**]. This thing is the explanation for more frequently detection of mitosis with normal karyotype in patients with chronic lymphocytic leukemia. The researchers used *restriction fragment length polymorphism *(RFLP) of genes located on the long arm of chromosome 12 to demonstrate that Trisomy 12 is composed by the duplication of one of chromosomes 12 and by tripling one of the chromosomes, with the loss of the other. In addition, using RFLP technology type, researchers have demonstrated that this chromosomal abnormality is found in all malignant cells [**32**].

Approximately 10% to 20% of patients may experience leukemic cells with deletions in the long arm of chromosome 11 marked 11q - [**19,33,34**]. It was observed that these patients are younger (under 55 years old) and they have the disease forms of aggressive evolution. 11q- deletions were identified, with critical points on 11q21, 11q22 and 11q14 levels [**32**].

A chromosomal abnormality intensively studied in trials that include patients with CLL and met in a percentage of 5% -8% from newly diagnosed cases [**35**] is 17p- deletion determined by in situ hybridization method (FISH). This chromosomal abnormality is the deletion of short arm “p” of chromosome 17, and the increased importance of this mutation is the fact that p53 gene - considered a “guardian of the genome” has its locus at 17p level. Among the multiple functions of p53 is that one to preserve the integrity and proper functioning of gene transmission and involvement in the self-destruction of cells with genetic defect. 17p deletion is associated with a poor/reserved prognosis, the most therapeutic regimens used in the treatment of CLL involving proper function of p53 [**1,2,36**]. In the analysis performed only Alemtuzumab and high-dose corticosteroids may lead to destruction of malignant cells in the presence CLL chromosome mutations involving p53 deficiency [**2,37,38**].

There are, however, cases where the progress of patients with 17p deletion is favorable. In these patients, was detected the presence of mutations in the VH genes. Cells with mutations in the VH genes levels have a slower rate of multiplication and even in the situation of p53 deficiency, the disease evolution and aggressiveness is greatly reduced, and the response to treatment is encouraging.

**Minimal residual disease**. The initiating treatment moments, according to prognostic factors identification for diagnosis, as well as the toxicity of various regimens, alter CLL evolution. Nevertheless, the final goal is to achieve a long-term complete remission. An increasing importance has to determine minimal residual disease. Flow cytometry and real-time quantitative PCR represent the new techniques and of high accuracy in the detection of minimal residual disease [**39**].

Tests can be performed both from peripheral blood and from bone marrow aspirate, the latest version being preferred in patients whose treatment regimens have been used monoclonal antibodies. It is still questionable usefulness of further targeted therapies for patients with presented minimal residual disease, although it has been demonstrated that their prognosis is more reserved and greater risk of relapse. Presence of more than a CLL cell to 10,000 leukocytes, define the absence of minimal residual disease and is an extremely important positive prognostic factor [**13**].

### Conclusions

Chronic lymphocytic leukemia, despite being still considered an indolent lymphoproliferation, does not have a radical solution therapy. Most patients achieved a long-term complete remission, but it is still maintaining a significant percentage of patients whose evolution is complicated and whose response to treatment is unsatisfactory. The improvement of the laboratory techniques resulted in accurate identification of prognostic factors, helping the emergence of new tailored and targeted therapies. In CLL, the clinical heterogeneity of the disease is closely interlinked with complex molecular heterogeneity. Involvement of old and new prognostic markers will remain in continuous research, providing new insights into the mechanisms that contribute to the pathogenesis of CLL and to explore therapeutic agents that increase survival expectancy of patients with the high risk.
